# Analysis of Tribo-Charging during Powder Spreading in Selective Laser Sintering: Assessment of Polyamide 12 Powder Ageing Effects on Charging Behavior

**DOI:** 10.3390/polym11040609

**Published:** 2019-04-03

**Authors:** Nicolas Hesse, Maximilian A. Dechet, Juan S. Gómez Bonilla, Christian Lübbert, Stephan Roth, Andreas Bück, Jochen Schmidt, Wolfgang Peukert

**Affiliations:** 1 Institute of Particle Technology (LFG), Friedrich-Alexander-Universität Erlangen-Nürnberg (FAU), Cauerstraße 4, D-91058 Erlangen, Germany; nicolas.hesse@fau.de (N.H.); maximilian.dechet@fau.de (M.A.D.); juan.s.gomez@fau.de (J.S.G.B.); christian.luebbert@fau.de (C.L.); andreas.bueck@fau.de (A.B.); jochen.schmidt@fau.de (J.S.); 2 Interdisciplinary Center for Functional Particle Systems, Friedrich-Alexander-Universität Erlangen-Nürnberg, Haberstraße 9a, D-91058 Erlangen, Germany; 3 Collaborative Research Center 814—Additive Manufacturing, Friedrich-Alexander-Universität Erlangen-Nürnberg (FAU), Am Weichselgarten 9, D-91058 Erlangen, Germany; s.roth@blz.org; 4 Bayerisches Laserzentrum GmbH (blz), Konrad-Zuse-Straße 2-6, D-91052 Erlangen, Germany

**Keywords:** additive manufacturing, selective laser sintering, electrostatics, polymer ageing, polyamide 12, powder layer formation, surface potential, charge distribution, tribo-charging

## Abstract

Powder flowability is key to achieving high process stability and part quality by application of smooth and dense layers in selective laser sintering (SLS). This study sheds light on the rarely investigated effect of tribo-electric charge build-up during powder delivery in the SLS process. This is achieved by a novel approach to quantify electrostatic potentials during doctor blading. The presented model setup is used in combination with charge spectrometry and impedance spectroscopy to investigate the alterations in tribo-electric charging behavior for the most commonly used laser sintering material polyamide 12 in its virgin and aged, c.f. reused, states. We show that the electrostatic charge build-up is significantly enhanced for aged polymer powder material, likely contributing to altered performance in SLS processing.

## 1. Introduction

The term Additive Manufacturing (AM) summarizes a variety of techniques that allow the manufacturing of parts from viscous, liquid, powdered or layered materials without the use of any tools or molds. AM enables easy individualization of parts and offers high freedom of design unmatched by any conventional manufacturing method, thus making it ideal and superior to subtractive methods for the manufacture of complex parts at low piece numbers [1]. AM technologies incorporate various polymer processing techniques. Among them, fused filament fabrication (FFF), stereolithography (SLA) and binder jetting have become feasible in recent years and allow the production of parts with a wide variety of properties. However, the mechanical part properties still have to be improved for high loads [2]. One AM technique capable of producing dense parts with superior mechanical performance, thus favored in industrial environment is the powder-based selective laser sintering process (SLS) [3], in which components are built in a layer-by-layer fashion. Each consecutive layer is heated to a temperature closely below the melting point of the used polymer and then melted by a laser in accordance to the desired structural design [4,5]. Typically, semi-crystalline thermoplastic powders with a narrow size distribution and a mean diameter of about 50 µm are deposited by means of blade or roller coating to form a layer with a thickness of roughly 100 µm [5,6]. It is pivotal that the powder layers exhibit a smooth surface and a high packing density to ensure low part porosity. Hence, good powder flowability is key to achieve high and homogeneous production quality. Powder flowability and packing density can be tailored by classification of the powders (c.f. removal of fines), shape control (thermal and mechanical rounding) [7,8] and application of flowing aids. Additionally, the diversity of applicable polymers is limited by the stringent requirements on the material’s thermal, chemical and rheological properties like the sintering window [9], melt viscosity [10], isothermal crystallization [11] or ageing behavior [12,13]. While there is a variety of available materials, such as polystyrene (PS), poly (methyl methacrylate) (PMMA), polypropylene (PP), high-density polyethylene (HDPE), polyaryletherketones (PEAK), polyamide 11 (PA11) [14] and polyamide 6 (PA6), the overwhelming share in the market is occupied by polyamide 12 (PA12), which does not only exhibit good powder but also excellent thermal properties.

The factors influencing the powder-rheological behavior are manifold, ranging from morphological conditions like particle size distribution, shape or roughness to the physicochemical material properties determining the interparticulate interactions. Therefore, knowledge of the powder conditions during layer deposition is crucial to gain a better understanding of the underlying principles. One aspect often neglected and not yet fully understood is the role of electrostatic charge build-up during polymer powder handling operations resulting from prolonged contact, collision or friction. Both homogeneous and inhomogeneous material interactions may result in charge separation on the involved species. For the explanation of these effects, three complementary concepts are commonly used, i.e., the electron, ion and material transfer model. A comprehensive overview and deeper insights into the governing mechanisms resulting in triboelectric charging is given e.g., by Matsusaka et al. [15] and Lacks et al. [16].

The concept of electron transfer is well-established and extensively reviewed for the interaction of metals with non-oxidized surfaces. The triboelectric charging of metals is usually not critical in technical applications due to the high conductivity of involved species and, therefore, the fast transport of charges away from the contacting area. This behavior allows for an easy measurement of the transferred charges, which are considered to be a result from electron transfer processes. The amount of charge transferred is dependent on the contact potential difference arising from different work functions of the contacting metals [15,17]. Modifications to this model have been made to incorporate insulator contacts by assigning an effective work function to them or utilizing the concept of higher energy level surface electrons, so-called surface states. While these theories yield good agreement for some experimental observations, there are cases where they do not hold (see [15] and references therein).

Ion transfer as a reason for triboelectric charging is considered when weakly bound or mobile ions are involved in material contact. One prevalent example is the thin water layer adsorbed on many surfaces that can be responsible for exchanging ionic species during contact events [17].

Material transfer from one body to another can arise from impact, adhesion or friction between them and it can include charge transfer if the transferred material carries any charge. It has been shown that polymer particles impacting a metal wall generate a current by leaving behind material on the wall [18]. Charge transfer between adhering and separated polymer particles can be assessed by AFM measurements whereby the single particle charge can be determined from the force-separation curve [19]. In the case of highly functionalized particles, like those used in SLS a material transfer of flow aids or other additives is conceivable.

While there is significant research on the generalized principles of contact electrification of powders for changing basic conditions like humidity [20], the influence of triboelectric charging for polymers in varying reactor designs [21,22] and experimental set-ups to replicate reactor or fluidized bed conditions [23], the investigation of triboelectric phenomena during deposition processes used in additive manufacturing remains elusive.

This study’s aim is introducing a method and a model setup to quantify the electrostatic potentials during powder spreading by means of blade coating. Moreover, the technique represents a novel way for an in-line measurement and controlling system for powder application strategies that rely on electrostatic interactions, such as an electrophotographic multi-material powder deposition [24]. The applicability of the model setup is demonstrated for polyamide laser sintering powders. It is utilized to examine the triboelectric behavior of virgin and aged laser sintering powder. Due to the elevated temperatures during processing, polymer powders can exhibit altered properties even though they are not used for part generation [25,26]. Especially polyamides suffer from high temperatures because of solid-state post-condensation, resulting in a prolonged molecular chain length [12,13,27]. For economic reasons these aged powders are reused by mixing with virgin material. The effects leading to changes in powder flowability for these powders are not yet sufficiently understood and will be discussed on the basis of measurements with the aforementioned set-up and by means of charge to particle size ratios obtained by charge spectrometry for both, virgin and used PA12 powders.

## 2. Materials and Methods

### 2.1. Materials

The polymer material used in this study is a commercially available polyamide 12 (PA12) laser sintering powder (PA2200, EOS GmbH, Krailling, Germany). Two kinds of conditioning were investigated, the virgin and an aged powder state, which has gone through three consecutive building processes in a FORMIGA P110 system (EOS GmbH, Krailling, Germany), each having a building time of 15.5 h, resulting in a cumulated building time of 46.5 h. This does not consider the heating-up and unregulated cooling-down phases. Powder has not been refreshed between processes. The building chamber temperature applied was 168 °C and the withdrawal chamber temperature was set to 150 °C. In each process, tensile strength bars and charpy test specimen have been built with a volumetric filling degree of 7.4% of the building chamber.

### 2.2. Laser Diffraction

Particle size distributions have been determined by laser diffraction (Mastersizer 2000, Malvern Panalytical Ltd., Kassel, Germany). For aerosol generation a dry dispersion unit (Scirocco 2000, Malvern Panalytical Ltd., Kassel, Germany) was operated at a pressure of two bar. The values given in this paper are calculated by averaging over five consecutive measurements. For evaluation, a Rayleigh-Mie model assuming spherical particles is used.

### 2.3. Impedance Spectroscopy

Impedance analyses have been carried out using the dielectric spectrometer Alpha-Analyzer (Novocontrol, Montabaur, Germany) equipped with a ZGS head. Prior to measurements, samples have been prepared by pelletising at 300 MPa pressure (MP150, Maassen GmbH, Reutlingen, Germany) with a 25-millimetre diameter press die set. For each pellet 600 mg of polymer powder were used, resulting in a thickness of 1.4 mm. The pellet samples have been mounted between two disc-shaped electrodes connected to the impedance analyser. Measurements have been performed at ambient conditions (19 °C and 30% relative humidity) in the frequency range 10^−2^ Hz to 20 MHz at 1 Volt root mean square (Vrms).

### 2.4. Powder Spreader Integrated Voltmeter Device

**Figure 1:**
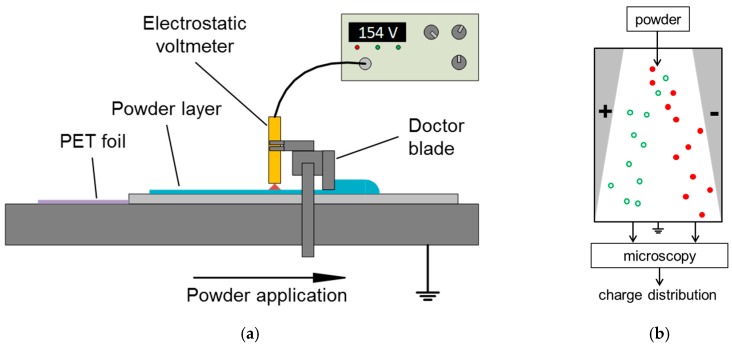
(**a**) Schematic depiction of the model powder spreading setup with the electrostatic voltmeter for measuring a powder’s surface potential during powder spreading. (**b**) Schematic functionality of the q/d-meter.

A novel lab-scale device for the investigation of electrostatic charge build-up on laser sintering powders during powder deposition is depicted in Figure 1a. The set-up consists of an automated film applicator (Coatmaster 510, Erichsen GmbH & Co. KG, Hemer, Germany) positioned inside a glove box (SICCO Vitrum, Bohlender GmbH, Grünsfeld, Germany) to allow modifications in relative humidity or oxygen concentration of the ambient atmosphere. Due to the enhancement of the conductivity, adsorption of water on the surface can alter the electric properties of polymer particles drastically [17]. Powder spreading is done by a doctor blade (Multicator 411, Erichsen GmbH & Co. KG, Grünsfeld, Germany), which allows the stageless variation of gap height between 1 µm and 1000 µm with an accuracy of 1 µm by a micrometer screw. Restrictions to the resulting powder layer thickness can apply according to the particle sizes of the used material. The deposition speed can be varied between 0.1 mm/s and 100 mm/s, enabling the reproduction of typical coating speeds applied in SLS. The film applicator incorporates a heatable suction plate for fixation of the grounded aluminum base plate on which the experiments are conducted. On the traverse of the applicator, an electrostatic voltmeter probe (model 1017AE, Monroe Electronics Inc., Lyndonville, NY, USA) is mounted. The probe is connected to an electrostatic voltmeter (ISOPROBE model 244A, Monroe Electronics Inc., Lyndonville, NY, USA) to measure the surface potential of the applied powder layer in a range between ±3000 V. Research has shown that surface potentials measured via voltmeter correlate well with the surface charge density of polymer samples examined in suitable Faraday-cup arrangements [28], thus enabling charge quantification. The relationship between potential difference *Δφ* and surface charge density *σ_E_* is given by Equation (1):

(1) $$\Delta \varphi \;=\;\frac{\sigma_{E}\cdot D}{\varepsilon_{0}\cdot \varepsilon_{r}}$$

where, *D* is the layer thickness of the dielectric and *ε_r_* and *ε*_0_ are the relative and the vacuum permittivity, respectively. Equation (1) is valid for a solid material film and does not consider the spherical character of the particulate bulk material. Since charges are generated at the layer surface and in the bulk material by different mechanisms their exact location is not known and therefore it is not possible to make assumptions about the charge distribution along the powder layer height based on the measured data.

The response time of the used voltmeter device is less than three milliseconds and therefore enables measurements at high powder spreading speeds. However, the speed is limited by the sampling rate of data recording, which is done at a frequency of 5 Hz via LabVIEW (National Instruments, Austin, TX, USA). Instruments have been calibrated prior to the experiment to exhibit no potential difference between the probe and the grounded base plate. Due to the proximity to the doctor blading fixture, the measurement of the electrostatic surface potential occurs approximately four centimeters behind the layer formation, lowering the potential influence of charge relaxation processes. Depending on the probe to surface spacing, the measured signal is a mean value representative for a circular projection area on the powder bed of varying size. Measurements were performed with a constant probe to surface spacing corresponding to a spot size at the powder bed surface of approximately six millimeters in diameter. To identify the beginning of the applied powder layer precisely, a polyethylene terephthalate (PET) foil was positioned in front of the grounded metal plate. The foil was triboelectrically charged manually to exhibit a strong negative charge. This allows an easy detection of the beginning of the positively charged polyamide powder layer by observing the zero crossing in the measurement files. Measurements over extended periods at zero potential have shown, that the accuracy of the instrumental setup is limited by the recording of the voltmeters output signal. The standard deviation of the detected signal noise is 0.34 V with a maximum deviation of ±0.97 V. Typically, the relative measuring error of voltmeter devices increases at low input signals, therefore the given values are considered as worst-case scenario.

### 2.5. Charge Spectrometry

Charge (q) to particle size (d) ratios are investigated by means of charge spectrometry (q/d-meter, Epping GmbH, Neufahrn bei Freising, Germany), schematically visualized in Figure 1b. The device uses low pressure to accelerate a pulsed particulate stream into an electric field with static field strength. According to Equation (2):

(2) $${\vec{F}}_{el}\;=\;q\cdot \vec{E}$$

where a charged particle’s trajectory is diverted by *F_el_*, the force the electric field exerts on it. Here, *q* represents the charge of the particle and *E* the strength of the electric field. The force resisting this diversion is the drag force

(3) $$F_{d}\;=\;\frac{\rho_{F}}{2}\left|{\vec{v}}_{rel}\right|{\vec{v}}_{rel}\frac{\pi }{4}d^{2}c_{d}$$

where *v_rel_* is the particle’s velocity relative to the surrounding fluid of density *ρ_F_*, *c_d_* is the Reynolds number dependent drag coefficient and d is the particle’s diameter. By balancing Equations (2) and (3) and gravity, particle trajectories can be calculated by integrating the balance of forces for their non-stationary movement. According to their movement pattern and polarity, particles are deposited on specimen slides that are mounted in front of the field-generating capacitor plates. By optically analyzing the slides with a scanning camera system, the projection area of the particles can be determined. Therefore, it is possible to calculate their charge-to-diameter ratio (q/d) from the position on the slides when flow conditions and electric field strength are known. Before any measurement, samples are mixed with magnetic iron carrier particles and activated by vortex mixing for 10 s at 2500 rpm (Digital Vortex Mixer model 945312, VWR international BVBA, Leuven, Belgium), resulting in more defined q/d-distributions than omitting powder preparation. The iron particles are held back in the sample container by a magnetic field and therefore do not get deposited on the specimen slides. Each measurement has been performed three times with several thousand particles detected in each measurement to guarantee statistical relevant data. A more exhaustive description of the concept of the measurement device and its unique selling point, the used free air beam technique, can be found in Epping et al. [29].

Particle size distributions are inherently measured by determining the q/d ratio of deposited polymer particles via charge spectrometry. Sizes are obtained by counting the number of pixels obscured by a charged particle on a specimen slide and their diameter is calculated by assuming round spheres, resulting in the same simplification of the particles morphology as the laser diffraction measurements.

## 3. Results and Discussion

### 3.1. Powder Spreader Integrated Voltmeter Device

**Figure 2:**
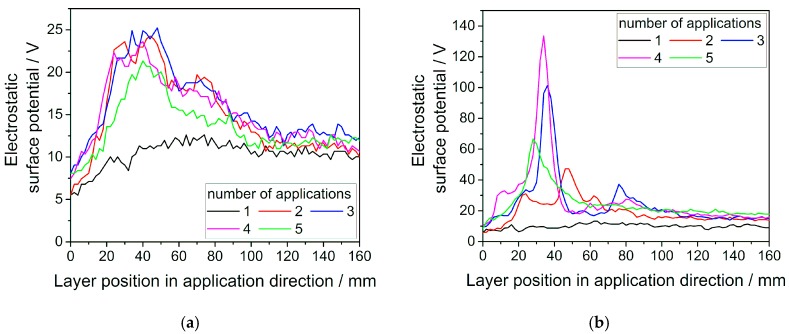
Electrostatic surface potential for (**a**) virgin and (**b**) used PA12 laser sintering powder during layer deposition for repeated application of the same powder sample with a velocity of 10 mm/s. Measurements have been performed at 30% relative humidity.

Figure 2 illustrates the progression of the electrostatic surface potential along the direction of layer deposition. Both virgin Figure 2a and used Figure 2b powders have been investigated. The same sample of PA12 laser sintering powder has been deposited and measured multiple times with an application speed of 10 mm/s.

The first application yields lower potentials for both kinds of ageing states with rather steady trends along the direction of powder deposition, whereas consecutive experiments exhibit elevated values and a clear maximum within the first 50 mm of the powder layer. Single particle contacting experiments with atomic force microscopy (AFM) have shown that the amount of charge transferred by contact electrification correlates well with the number of contacts for a variety of insulating materials like polystyrene (PS) and polydimethylsiloxane (PDMS) [30,31,32]. Similar experiments conducted with polymer tips contacting a plain surface on a Kelvin probe force microscopy (KPFM) system do also show an influence of the force involved in frictional contact, with a higher force leading to increases in charge generation [33]. According to these observations, the elevated values of the surface potential at earlier powder layer positions can be attributed to the intensified contacting conditions during the start of the doctor blading process. Since the powder reservoir in front of the applicator diminishes with increasing path length, both, the number of contacts and the forces acting onto single particles decrease over the course of layer application. While there seems to be a quickly reached saturation limit for the surface potential of repeatedly applied virgin powder, this indication is much less explicit for aged powder. Overall, the potentials measured for aged powders exceed those of the virgin powder considerably.

Whilst virgin powder has a peak potential of approximately 25 V, the aged material reaches values up to almost 140 V in this experiment. Possible reasons for this could be the depletion or degradation of an antistatic agent in the powder material or a change of electrical properties due to alterations in the molecular structure of the polymer by solid-state post-condensation. The main factor influencing charge build-up is the surface resistivity, since good conductivity results in a fast equilibration of charges [17,34]. It is known that polymer swelling and adsorption of ambient water have a strong influence on the surface resistivity and therefore change electrical properties of polyamides [20]. Consequently, a changed water absorption behavior due to alterations in the molecular structure could also explain the observed results. This is in line with the impedance spectroscopy measurements shown in Table 1 that exhibit a reduced conductivity for aged powder, especially at low frequencies.

**Figure 3:**
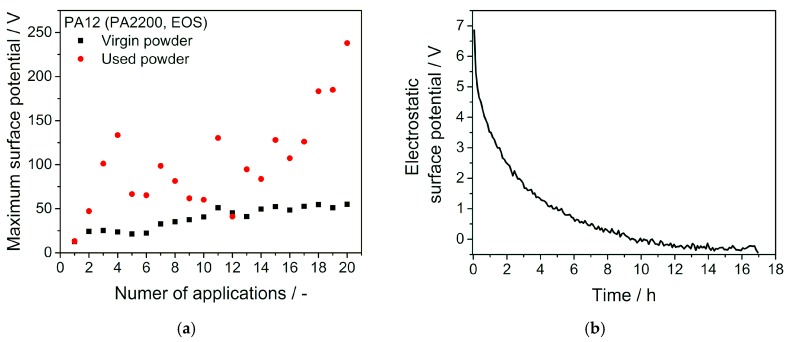
(**a**) Maximum surface potential measured for a high number of repeated applications of virgin and used PA12 powders. (**b**) Relaxation of the electrostatic surface potential over time. Voltmeter probe has not been moved relatively to the powder layer in this measurement. Experiment has been conducted at 30% relative humidity.

Moreover, the data displayed in Figure 2 shows that multiple applications of the same powder in close temporal proximity should be avoided when aiming for a lower influence of electrostatics during powder layer formation. This result is apparent for both, virgin and used powder and is clarified in Figure 3a by means of the maximum surface potential measured for up to 20 repetitions of the spreading process. Since electrostatic-related defects in powder layer are presumed to be most likely found at highly charged positions in the powder bed, the most critical value in this process mimicking measurement is the highest occurring surface potential. While there seems to be a converging value for virgin powder, the used powder with altered properties does not reach a limiting value of maximum surface potential within a reasonable amount of repeated powder spreading operations. Repeated applications are of importance for many simpler laser sintering machines like the one used for powder aging experiments in this work. It uses a blade-based powder spreader that applies a certain amount of powder, which is not exactly equal to the amount of powder needed for a single layer. Therefore, the surplus powder is spread multiple times while being exerted to triboelectric charging conditions in each spreading process. Eventually, new powder will be applied to the remainder of the powder that has already been spread several times. However, machines that are more sophisticated use an overflow mechanism that removes surplus powder from the building chamber after being applied once. In this case, the relevant measurement is the first application of powder which can be considered less critical in comparison to repeated applications as the provided data shows.

In Figure 3b the charge relaxation behavior is depicted for a spatially static measurement over several hours. Analogue to earlier experiments the layer formation was done at a constant powder deposition speed of 10 mm/s for homogeneous layer formation. The observed surface potential decays exponentially and reaches an uncharged state after approximately 12 h. Therefore, the relaxation times of triboelectric charges are well beyond the times between consecutive layer applications in the laser sintering process, where a new layer is applied every few minutes.

The influence of charge build-up in the context of SLS is seen rather disruptive. In theory, particles with a repelling net charge could have a positive effect on powder flowability by reducing attractive interparticulate forces that lead to particles sticking together and therefore a reduced flowability. However, this will also lead to worse bulk densities in the polymer powder layer, which can be associated with a higher porosity and impaired mechanical properties of the finished parts [35]. Another detrimental effect that is associated with electrostatically charged particles is their deposition on exposed parts in the system [22], leading to high post-processing efforts and electrical fields that are capable to interfere with internal sensors and instruments [36]. Instead of using Coulomb forces to prevent adhesive, short-range interactions, the commonly used and better approach is minimizing the van der Waals force by coating surfaces with nanoparticles that act as spacer [37].

### 3.2. Charge Spectrometry

**Figure 4:**
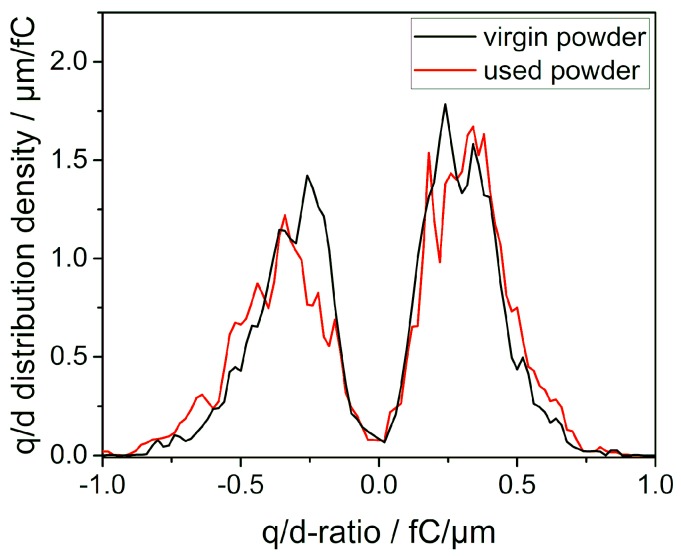
Charge-to-diameter distributions of virgin and used PA12 laser sintering powder obtained by charge spectrometry measurements. Shown values are averaged over three measurements.

Since the movement pattern of a particle in an electric field can be influenced either by its charge or by its size, both quantities need to be accounted for. Therefore, the charge to diameter (q/d) ratio distributions are used when looking at charge spectrometry data. Figure 4 depicts the bipolar charge distributions for virgin and used PA12 which can be described by two Gaussian distributions. The gap around a q/d-ratio of zero femtocoulomb per micrometre might still include particles which do not get detected due to the insufficient curvature of their trajectories resulting from their low q/d-ratios. While it is possible to detect significant alterations in the voltmeter measurements for virgin and aged polymers, only minor changes can be resolved by charge spectrometry. Table 2 shows the mean q/d values of both the positively and the negatively charged share of virgin and used powders as well as the percentage of particles with a positive polarity. The calculated mean q/d values are slightly higher for used laser sintering powder in comparison to the virgin powder, which is in line with the observed surface potential in the powder spreading model experiment for the two types of powders. Additionally the shift to a higher amount of positively charged particles is in good agreement with not only the observed positive potentials from voltmeter measurements but also the empirical and well-established triboelectric series which predicts a small net positive charge for polyamides [16,38]. Considering the measured value of ±0.33 fC/µm a single particle with a diameter of 50 µm possesses an average of ~10^5^ elementary charges.

**Figure 5:**
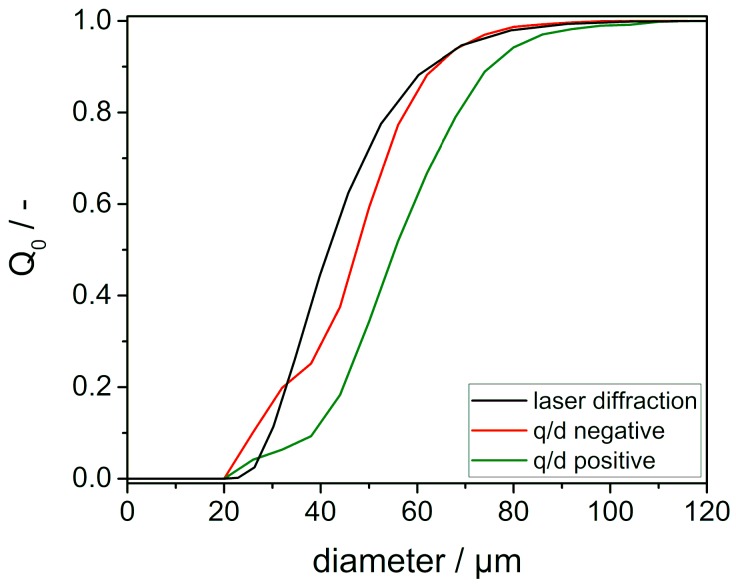
Number weighted sum distribution of particles above 20 µm detected in charge spectrometry (red and green) and in laser diffraction measurements (black) of virgin PA12 powder. Shown data is averaged over three (q/d), respectively five (laser diffraction) measurements.

Negatively charged particles exhibit a smaller particle size than their positive counterparts as shown in Figure 5. Here, the number weighted sum distribution (Q_0_) of particles deposited in the electric field detected by the charge spectrometer is shown for virgin PA12. Since the data in Table 2 indicates no differences in q/d-ratio for opposing polarities, the shift in the diameter distribution necessitates an equally higher charge on positive particles. While it cannot be ruled out that this observation is based on inhomogeneous material contact conditions arising from e.g., flow aids coating the surface of the particles, we attribute it to the phenomenon of asymmetric contact area electrification. The reason behind tribo-charging in homogeneous material contacts remains unclear since there is no material specific driving force for electron transfer. However, one aspect frequently observed shows that smaller particles tend to charge negatively and larger ones positively due to trapped non-equilibrium, high energy surface states. While electrons in high energy states cannot equilibrate to lower states on the same surface, they can transfer by collision with other particles. It has been shown that collision dynamics lead to a net transfer of high energy states from large particles to smaller ones, leading to smaller particles being charged negatively [39,40,41,42].

Table 3 lists the characteristic particle sizes x_10,0_, x_50,0_ and x_90,0_ for virgin and used PA12 powder measured by either laser diffraction or charge spectrometry. While there is no significant change for the virgin material when compared to the aged one when looking at the laser diffraction measurements, there is a difference for the q/d-meter data. Even though several thousands of particles were detected, this might not be sufficient when looking at specific size fractions to ensure sufficient statistical agreement. Another important aspect is that not the complete particle ensemble is analysed since weakly charged particles may not get deposited during measurement. Therefore, most of those particles leave the measurement cell without being detected, resulting in an overestimation of the obtained size distribution.

## 4. Conclusions

In this work, an innovative setup for the analysis of electrostatic surface potential build-up, during a powder spreading process was presented. The combination of the powder delivery process during SLS and with an electrostatic surface potential measurement leads to novel insights into the in-situ triboelectric charge build-up during powder application. The presented measurement principle can easily be transferred and integrated into an SLS machine to produce real time data at building chamber temperature for monitoring of critical layer formation processes during development of new powder systems or to monitor the spatial distribution of surface potential and thus, surface charge in the building area. Moreover, by applying our set-up for the analysis of virgin and aged PA12 powder, we could show for the first time that ageing also affects the charging behavior of the powder material. The differences between virgin and aged powder material in impedance (c.f. smaller conductivity of aged powder) and charge spectroscopy measurements (c.f. larger mean q/d ratio for aged material) are enhanced when looking at the integral behavior of the bulk material under processing conditions (c.f. higher surface potentials for tribo-charging of aged powder as compared to virgin powder). Effects for less optimized materials in earlier development stages are expected to be even more incisive. Whereas mostly exclusively flowability and viscosity, respectively molecular weight distribution, were assessed in terms of ageing so far, we could show that also significant changes in triboelectric properties during powder ageing occur. These have to be taken into consideration for the development of powder recycling strategies and the assessment of powder reusability.

## Figures and Tables

**Table 1:** Mean conductivities obtained by impedance spectroscopy measurements of virgin and aged PA12 for varying frequencies. Values in brackets represent the standard deviation over five measurements.

Material	Conductivity at 10^−2^ Hz [×10^−14^ S/cm]	Conductivity at ~10^2^ Hz [×10^−10^ S/cm]	Conductivity at 10^7^ Hz [×10^−5^ S/cm]
Virgin powder	2.22 (±0.13)	1.47 (±0.06)	2.65 (±0.08)
Used powder	1.55 (±0.06)	1.31 (±0.05)	2.43 (±0.04)

**Table 2:** Mean q/d values for positive and negative charged particles and share of positive polarity particles obtained by q/d-meter measurement of virgin and aged PA12. Values in brackets represent the standard deviation over three measurements.

Material	Mean q/d Value Positive [fC/µm]	Mean q/d Value Negative [fC/µm]	Share of Positive Polarity Particles [%]
Virgin powder	+0.33 (±0.02)	−0.33 (±0.02)	55.8 (±3.7)
Used powder	+0.35 (±0.01)	−0.38 (±0.01)	57.9 (±1.5)

**Table 3:** Characteristic particle sizes of PA12 laser sintering powders for different ageing states and measurement methods.

Method	Material	x_10,0_	x_50,0_	x_90,0_
laser diffraction	Virgin powder	30 µm	42 µm	62 µm
laser diffraction	Used powder	30 µm	42 µm	63 µm
q/d-meter	Virgin powder	28 µm	51 µm	71 µm
q/d-meter	Used powder	26 µm	48 µm	67 µm
